# Prognostic Value of Erythroblastic Leukemia Viral Oncogene Homolog 2 and Neuregulin 4 in Hepatocellular Carcinoma

**DOI:** 10.3390/cancers15092634

**Published:** 2023-05-06

**Authors:** Woo Sun Rou, Hyuk Soo Eun, Sorim Choung, Hong Jae Jeon, Jong Seok Joo, Sun Hyung Kang, Eaum Seok Lee, Seok Hyun Kim, In Sun Kwon, Bon Jeong Ku, Byung Seok Lee

**Affiliations:** 1 Department of Internal Medicine, Chungnam National University College of Medicine, Daejeon 35015, Republic of Korea; rws00@cnuh.co.kr (W.S.R.); hyuksoo@cnuh.co.kr (H.S.E.); kynik@cnuh.co.kr (H.J.J.); jujngs@cnuh.co.kr (J.S.J.); porrtos@cnuh.co.kr (S.H.K.); leeusgi@cnuh.co.kr (E.S.L.); midoctor@cnuh.co.kr (S.H.K.); 2 Division of Gastroenterology and Hepatology, Department of Internal Medicine, Chungnam National University Sejong Hospital, Sejong 30099, Republic of Korea; 3 Division of Gastroenterology and Hepatology, Department of Internal Medicine, Chungnam National University Hospital, Daejeon 35015, Republic of Korea; 4 Department of Medical Science, Chungnam National University College of Medicine, Daejeon 35015, Republic of Korea; thfla79@cnu.ac.kr; 5 Clinical Trial Center, Statistics Office, Biomedical Research Institute, Chungnam National University Hospital, Daejeon 35015, Republic of Korea; 6 Division of Endocrinology, Department of Internal Medicine, Chungnam National University Hospital, Daejeon 35015, Republic of Korea

**Keywords:** hepatocellular carcinoma, erythroblastic leukemia viral oncogene homolog 2 (ERBB2), neuregulin 4 (NRG4), mitogen-inducible gene 6 (MIG6), biomarkers

## Abstract

**Simple Summary:**

One of the great advantages of serum biomarkers is that they can be easily obtained in the form of a liquid biopsy of the patient’s blood, which is minimally invasive and allows for repeatable measurements. In particular, molecular biomarkers from signaling pathways provide information on tumor characteristics and heterogeneous molecular profiles and enable us to predict prognosis and develop a rationale for therapeutic strategies. The erythroblastic leukemia viral oncogene homolog (ERBB) family has been implicated in hepatocarcinogenesis and is associated with a worse prognosis in hepatocellular carcinoma (HCC). However, its role as a serum biomarker has not been fully evaluated. In the present study, we revealed that serum ERBB2 and neuregulin 4 (NGR4) are independent prognostic factors for survival and tumor recurrence and suggested a possible synergistic effect between these two prognostic factors. Our study could provide predictive biomarkers for evaluating HCC prognosis and monitoring treatment response in patients with HCC.

**Abstract:**

Although the roles of erythroblastic leukemia viral oncogene homolog 2 (ERBB2), neuregulin 4 (NRG4), and mitogen-inducible gene 6 (MIG6) in epidermal growth factor receptor signaling in hepatocellular carcinoma (HCC) and other malignancies have been previously investigated, the prognostic value of their serum levels in HCC remains undetermined. In the present study, correlations between serum levels and tumor characteristics, overall survival, and tumor recurrence were analyzed. Furthermore, the prognostic potential of the serum levels of these biomarkers was evaluated relative to that of alpha-fetoprotein. Both ERBB2 and NRG4 correlated with the Barcelona Clinic Liver Cancer stage, ERBB2 correlated with the tumor-maximal diameter, and NRG4 correlated with a tumor number. Cox proportional hazards regression analysis revealed that ERBB2 (hazard ratio [HR], 2.719; *p* = 0.007) was an independent prognostic factor for overall survival. Furthermore, ERBB2 (HR, 2.338; *p* = 0.002) and NRG4 (HR, 431.763; *p* = 0.001) were independent prognostic factors for tumor recurrence. The products of ERBB2 and NRG4 had a better area under the curve than alpha-fetoprotein for predicting 6-month, 1-year, 3-year, and 5-year mortality. Therefore, these factors could be used to evaluate prognosis and monitor treatment response in patients with HCC.

## 1. Introduction

Hepatocellular carcinoma (HCC) is a heterogeneous tumor with various risk factors, including chronic viral hepatitis B and C, excessive alcohol intake, and non-alcoholic fatty liver disease, which triggers hepatocellular injury, progressive destruction and aberrant regeneration [1,2]. Consequently, HCC results from numerous genetic and epigenetic alterations and considerable changes in cell signaling pathways that occur in hepatocytes [3].

Erythroblastic leukemia viral oncogene homolog 2 (ERBB2), a member of the epidermal growth factor receptor signaling pathway, plays a key role in cell proliferation, differentiation, and survival [4,5]. ERBB2 is overexpressed in some cancers, and its overexpression is associated with aggressive behavior and poor prognosis [6,7]. In patients with breast cancer, ERBB2 is a crucial biomarker for improving diagnostic accuracy and therapeutic outcomes [8]. Abnormal ERBB2 expression is associated with poor prognosis and tumor recurrence in patients with HCC [9,10,11]. However, measuring tissue expression of ERBB2 is challenging because HCC can be diagnosed based on typical radiological findings without histological examination. Additionally, 12–66% of HCCs exhibit intratumoral heterogeneity; thus, the entire cancer cannot often be represented by extracting a few tissues [12,13,14]. Serum biomarkers may overcome these difficulties as they are reproducible and easy to measure repeatedly during treatment [12]. Several studies have reported that serum ERBB2 is associated with breast cancer prognosis and can be used to monitor treatment responses [15,16]. However, the use of serum ERBB2 as a biomarker for HCC has not been investigated yet.

Here, we evaluated the prognosis of patients with HCC by measuring serum levels of ERBB2, which exhibits oncogenic activity as a major factor of ERBB receptors in the EGFR signaling pathway, and neuregulin 4 (NGR4), which has been evaluated in various carcinomas as a major ligand for ERBB4. Mitogen-inducible gene 6 (MIG6), which is involved in the feedback regulation of the EGFR pathway, was also measured in the blood of patients with HCC to assess its prognostic value. This study aimed to evaluate the relationship between serum ERBB2, NRG4, and MIG6 levels and tumor characteristics, overall survival, and tumor recurrence to determine the potential prognostic value of these signaling molecules for HCC.

## 2. Materials and Methods

### 2.1. Patients and Sample Collection

This study included patients diagnosed with HCC. The blood samples of the patients were stored in the Biobank of Chungnam National University Hospital, which is a member of the National Biobank of Korea. Among the blood samples stored from 14 May 2009 to 31 December 2019, those obtained from patients with HCC were randomly dispensed based on the modified Union for International Cancer Control stages at the time of blood sampling. Data pertaining to clinical, laboratory, and imaging variables, such as computed tomography (CT) and magnetic resonance imaging (MRI) findings, and treatment modalities were obtained from the medical records at the time of blood sampling.

### 2.2. Diagnosis of HCC

HCC was diagnosed via histological analysis using percutaneous biopsy or surgery or based on a distinctive radiological pattern of hyperenhancement in the arterial phase and washout in the portal venous or delayed phase on contrast-enhanced CT or MRI according to the American Association for the Study of Liver Diseases criteria [17].

### 2.3. Blood Sampling, Storage, and Measurement

Blood samples were collected by venipuncture into vacuum tubes. The serum was separated by centrifugation at 3000 rpm for 10 min at room temperature and then centrifuged again at 5000 rpm for 5 min to obtain cell-free serum. The samples were stored at −80 °C until measurement.

ERBB2, NRG4, and MIG6 were quantified using commercially available enzyme-linked immunosorbent assay (ELISA) kits and an automated sandwich immunoassay. Serum ERBB2 levels were analyzed using an ErbB 2 Human ELISA Kit (Abcam, Waltham, MA, USA; Product No. ab100510). Serum NRG4 levels were measured using a human ELISA kit for NRG4 (Cloud-clone Corp., Katy, TX, USA; Product No. SEC174Hu). Serum MIG6 levels were measured using a human ERRFI1 ELISA kit (FineTest, Wuhan, China; Product No. EH14434). The measurements were performed in accordance with the manufacturer’s instructions, ensuring quality control. All the samples were analyzed in duplicate.

### 2.4. Statistical Analysis

Differences in serum ERBB2, NRG4, and MIG6 levels according to patient and tumor characteristics were evaluated using independent t-tests and one-way analysis of variance for continuous and parametric variables. Meanwhile, the Mann–Whitney U test or Kruskal–Wallis test was used for continuous and nonparametric variables. The associations between the serum ERBB2, NRG4, MIG6, and alpha-fetoprotein (AFP) levels were assessed using Pearson’s correlation. The relationships between the serum ERBB2, NRG4, and MIG6 levels and ordinal variables were analyzed using Spearman’s correlation. To evaluate serum ERBB2, NRG4, and MIG6 levels as independent prognostic factors for overall survival and tumor recurrence, univariate and multivariate analyses were performed using Cox proportional hazards regression. Receiver operating characteristic curves and areas under the curve were compared to assess the prognostic value of serum ERBB2, NRG4, MIG6, and AFP levels to predict mortality. The data were analyzed using the Statistical Package for the Social Sciences (SPSS) version 26.0 (SPSS Inc., Chicago, IL, USA) and MedCalc statistical package version 19.5.3 (MedCalc, MariaKerke, Belgium). A *p*-value of <0.05 was considered statistically significant (* *p* < 0.05, ** *p* < 0.01).

## 3. Results

### 3.1. Baseline Characteristics of the Patients and Tumors

The median follow-up was 34.5 months (range, 0.8–119.1; interquartile range [IQR], 12.8–56.6). The mean patient age was 62.0 years, and 47 (78.3%) patients were men (Table 1). Twenty-seven patients (45.0%) were histologically diagnosed with HCC, and the remaining patients (55%) were diagnosed with HCC according to the noninvasive radiologic criteria based on the American Association for the Study of Liver Diseases guidelines. According to the Child–Pugh classification, the hepatic function was classified as class A in 48 patients (80.0%), class B in 8 patients (13.3%), and class C in 4 patients (6.7%). According to the Barcelona Clinic Liver Cancer (BCLC) staging system, 14 (23.3%), 20 (33.3%), 7 (11.7%), 15 (25.0%), and 4 (6.7%) patients had BCLC stage 0, A, B, C, and D HCCs, respectively. Of the patients, 31 (51.7%) and 29 (48.3%) had solitary and multiple lesions, respectively, with a maximal diameter of 4.0 ± 3.6 cm. Additionally, ten patients (16.7%) had concomitant portal vein tumor thrombus (PVTT). Fifty-three patients received more than one treatment modality, of whom forty-seven (78.3%) demonstrated a complete response (CR); all the patients who achieved a CR received a curative treatment such as resection or radiofrequency ablation. Of the patients who achieved a CR, HCC recurred in 29 (61.7%) patients during the observation period. The median time to HCC recurrence was 28.3 months (range, 1.9–94.0; IQR, 12.4–56.8).

### 3.2. Correlation among Serum ERBB2, NRG4, MIG6, and AFP Levels

ERBB2 and NRG4 demonstrated a weak correlation (Pearson’s r = 0.254, *p* = 0.05); however, no significant association was identified between the other factors. Furthermore, ERBB2, NRG4, and MIG6 serum levels were not significantly correlated with serum AFP levels (Pearson’s r = −0.078, *p* = 0.587 for ERBB2; r = 0.110, *p* = 0.443 for NRG4; and r = 0.007, *p* = 0.959 for MIG6).

### 3.3. Differences in Serum ERBB2, NRG4, and MIG6 Levels According to Tumor Characteristics

ERBB2, NRG4, and MIG6 serum levels were compared based on tumor characteristics, including BCLC stage, tumor-maximal diameter, and the number of tumors, using the Kruskal–Wallis test and bivariate (Spearman) correlation (Table 2). ERBB2 and NRG4 levels exhibited a moderate linear correlation with the BCLC stage (Spearman’s ρ = 0.386, *p* = 0.002 and ρ = 0.609, *p* < 0.001, respectively). ERBB2 demonstrated a moderate linear correlation with the maximal tumor diameter (Spearman’s ρ = 0.432, *p* = 0.001), and NRG4 demonstrated a moderate linear correlation with the number of tumors (Spearman’s ρ = 0.558, *p* < 0.001). MIG6 demonstrated a weak correlation with the BCLC stage (Spearman’s ρ = 0.281, *p* = 0.030).

### 3.4. Differences in ERBB2, NRG4, and MIG6 Serum Levels According to PVTT, Distant Metastasis, Liver Cirrhosis, Chronic Viral Hepatitis B or C, and Fatty Liver Statuses

Serum ERBB2, NRG4, and MIG6 levels were compared according to the presence of PVTT and distant metastasis (Table 3). Serum MIG6 (2.00 ng/mL vs. 0.65 ng/mL, *p* = 0.004), NRG4 (0.32 ng/mL vs. 0.17 ng/mL, *p* = 0.007), and ERBB2 (2.65 ng/mL vs. 1.50 ng/mL, *p* = 0.001) levels were higher in patients with PVTT than in those without PVTT. Patients with distant metastases had higher serum MIG6 levels than those without distant metastases (2.81 ng/mL vs. 0.66 ng/mL, *p* = 0.023). Serum NRG4 (0.16 ng/mL vs. 0.25 ng/mL, *p* = 0.003) and MIG6 (0.66 ng/mL vs. 1.56 ng/mL, *p* = 0.018) levels were lower in patients with an HBV infection than in those without an HBV infection. Serum MIG6 levels (2.81 ng/mL vs. 0.66 ng/mL, *p* = 0.012) were higher in patients with HCV infection than in those without HCV infection, whereas no significant differences for serum NRG4 (*p* = 0.093) and ERBB2 (*p* = 0.210) levels were observed. No significant differences in serum ERBB2, NRG4, and MIG6 levels were observed between the patients with and without fatty liver (*p* = 0.905, *p* = 0.275, and *p* = 0.427, respectively).

### 3.5. Predictors of Overall Survival and HCC Recurrence

The univariate analysis of survival demonstrated that HBV infection, Child–Pugh class, maximal tumor diameter, AFP, BCLC stage, treatment modality, ERBB2, and NRG4 were significant risk factors (Table 4). The multivariate analysis revealed that the serum ERBB2 level was an independent predictor of survival. Moreover, the Child–Pugh class, BCLC stage, and serum AFP level were independent predictors of survival. BCLC stage, ERBB2, and NRG4 were identified as significant risk factors for HCC recurrence in the univariate analysis (Table 5), whereas ERBB2 and NRG4 serum levels were independent predictors of HCC recurrence in the multivariate analysis.

### 3.6. Differences in Cumulative Survival and HCC Recurrence in Patients Grouped According to Serum ERBB2 and NRG4 Levels

**Figure 1:**
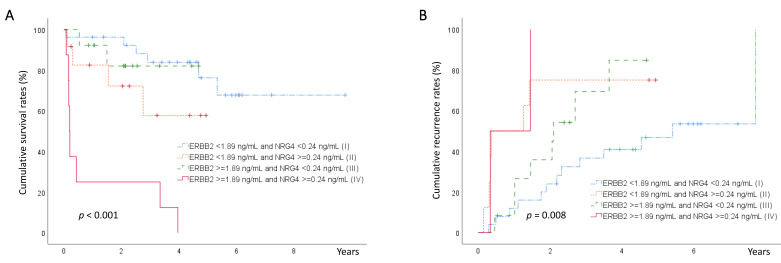
Kaplan–Meier curves for cumulative survival of the four groups, based on serum levels of ERBB2 and NRG4. (**A**) The group with elevated serum levels of both ERBB2 and NRG4 had a lower survival rate compared with that of the other groups (IV vs. II, *p* = 0.007; IV vs. I, III, *p* < 0.001). (**B**) Kaplan–Meier curves for cumulative recurrence for the four groups, based on serum levels of ERBB2 and NRG4. The group with elevated serum levels of both ERBB2 and NRG4 had a higher HCC recurrence compared with that of the groups with low serum NRG4 (IV vs. III, *p* = 0.030; IV vs. I, *p* = 0.002; IV vs. II, *p* = 0.840). (Abbreviations: ERBB2, erythroblastic leukemia viral oncogene homolog 2; NRG4, neuregulin 4; HCC, hepatocellular carcinoma).

Based on the mean serum levels of ERBB2 and NRG4, the samples were divided into four groups as follows: high serum levels of both ERBB2 and NRG4, high serum levels of ERBB2, and low serum levels of NRG4, low serum levels of ERBB2 and high serum levels of NRG4, and low serum levels of both ERBB2 and NRG4. Cumulative survival and HCC recurrence were compared between the groups. The cumulative survival rate was lower, whereas the recurrence rate was higher in the patients with high serum ERBB2 and NRG4 levels than in the other three groups (Figure 1).

### 3.7. Predictive Power of Serum ERBB2, NRG4, and MIG6 Levels for 6-Month, 1-Year, 3-Year, and 5-Year Mortality

**Figure 2:**
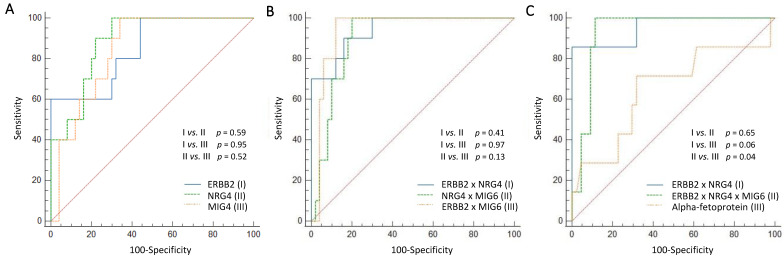
Graphs (**A**–**C**) present the receiver operating characteristic curves for the prediction of 6-month mortality. (**A**) The single factors, such as ERBB2, NRG4, and MIG6, were compared to each other, and (**B**) multiplied values were also compared. Although the comparison between single factors and the comparison between multiplied values did not show a significant difference, (**C**) the product of ERBB2 and NRG4 with MIG6 (AUC 0.940)/without MIG6 (AUC 0.942) showed superior results in AUC compared to the results for alpha-fetoprotein (AUC 0.727). (ERBB2, erythroblastic leukemia viral oncogene homolog 2; NRG4, neuregulin 4; MIG6, mitogen-inducible gene 6; AUC, area under the curve; red dotted line in the figure, random classifier).

Using the ROC curve analysis, the single factors, such as ERBB2, NRG4, and MIG6, were compared to each other (Figure 2A), and the ability of the multiplied values (Figure 2B,C) to predict 6-month (Table 6), 1-year, 3-year, and 5-year mortality were also compared (Table S1, Figure S1). The product of ERBB2 and NRG4 with or without MIG6 revealed a good AUC for predicting 6-month, 1-year, 3-year, and 5-year mortality, and predicting the 6-month mortality demonstrated the best AUC (Table 6, Figure 2C).

## 4. Discussion

Many mutations in oncogenes, tumor suppressor genes, and several important HCC-related signaling pathways have been identified. However, advances in the understanding of the molecular drivers of HCC have not yet been translated into biomarker-driven precision medicine trials [18]. AFP has been established as a biomarker for HCC diagnosis and prognosis. However, elevated serum AFP levels can also be observed in several other medical conditions, including acute and chronic hepatitis, cirrhosis, colitis, and germ cell tumors. PIVKA-II, another biomarker, is elevated in patients receiving warfarin or antibiotics and in those with alcoholic liver disease [19,20]. Therefore, there is an unmet need for new biomarkers. In this study, the serum levels of ERBB2 and NRG4, which are involved in the EGFR signaling pathway, correlated with tumor characteristics. Moreover, ERBB2 was an independent prognostic factor for the survival and recurrence of HCC, and NRG4 was an independent prognostic factor for HCC recurrence. The product of ERBB2 and NRG4, with or without MIG6, could predict 6-month, 1-year, 3-year, and 5-year mortality better than AFP.

The EGFR signaling pathway plays a key role in cell proliferation, differentiation, and survival by triggering downstream signaling pathways, such as Ras/Raf/mitogen-activated protein kinase, phosphatidylinositol-3-kinase/protein kinase B, and Janus kinase/signal transducer and activator of transcription, which are associated with the pathogenesis of tumors [21,22]. The EGFR receptor family comprises four members that belong to the ErbB lineage of proteins (ErbB1–4, also known as HER1–4) and function as homodimers or heterodimers [22]. Heterodimers containing ErbB2 exhibited robust and prolonged signaling activity [22,23,24]. ErbB2 may also mediate the oncogenic activity of ErbB4 receptor binding to a ligand [25]. For the formation of ErbB2/ErbB4 heterodimers, the ligands of the dimerization partner are essential. However, to date, no study has reported a ligand for ErbB2. Neuregulins are ErbB3 and ErbB4 ligands, and activated neuregulin-bound ErbB3, and ErBb4 can form heterodimers with ErbB2 [24,26].

ERBB2 expression in HCC tissues, based on immunohistochemical (IHC) staining, varies from 0% to 90% [9,27,28,29,30,31]. This variation in the results of IHC staining may be due to the intratumoral heterogeneity of HCCs [12,13] and differences in causative factors for HCC in different studies [11,32]. Several in vivo and in vitro studies have reported that ERBB2 is associated with liver cancer progression and epithelial–mesenchymal transitions [33,34,35]. In another study using the HCC dataset, ERBB2 overexpression was identified by analyzing ERBB2 mRNA amplification, which was related to the tumor stage in HCC samples. Trastuzumab, a monoclonal, anti-human ERBB2 protein antibody, inhibits tumor size and metastasis in vivo and in vitro through the upregulation of β-catenin and inhibition of SMAD3 [9]. Additionally, overexpression of ERBB2 is associated with poor prognosis in patients with HCC [9,10]. However, determining ERBB2 overexpression using IHC staining of tumor tissues requires performing a biopsy, and findings may be erroneous because of observer variability and non-standardized IHC assays and scoring systems. Moreover, repeated real-time follow-up is difficult using biopsies [36]. Using ERBB2 as a prognostic indicator for breast and gastric cancers, the most extensively studied cancers, several studies have demonstrated that tumor recurrence, metastasis, and poor overall survival are associated with high serum ERBB2 levels [15,16,37,38,39]. Although the tissue and serum levels of ERBB2 did not indicate any correlation, high serum levels of ERBB2 were associated with unfavorable prognoses [40,41]. Furthermore, some studies have reported that the serum ERBB2 level can be used to predict the response to anti-HER2 treatment [42,43]. In our study, higher serum ERBB2 levels were associated with an advanced BCLC stage, a larger tumor diameter, and the presence of PVTT. Furthermore, the serum ERBB2 level was demonstrated to be an important prognostic factor in predicting recurrence and survival, independent of tumor size and stage. This finding is consistent with those of recent studies that have reported poor tumor recurrence and survival in patients with HCC when ERBB2 was overexpressed in the tumor tissues [9,31].

ERBB2 functions physiologically as a heterodimer with other activated ERBB receptors. Even with the same heterodimer, receptor signaling differs depending on the type of ligand to which it binds, and qualitative differences exist [44,45,46]. NRG4, a ligand of the NRG family, binds only to ERBB4. NRG4 has been studied in breast and prostate cancers and has been reported to be associated with poor prognosis [26,47,48,49,50]. Our study revealed that the serum level of NRG4 was correlated with the BCLC stage and the number of HCC tumors. These findings are consistent with previous studies on breast and prostate cancers, which have demonstrated that increased NRG4 expression is observed at an advanced stage and in high-grade tumors [47,49]. NRG4 demonstrated a weak correlation with ERBB2; when both serum ERBB2 and NRG4 levels were high, the prognosis was unfavorable compared with the prognosis when either serum ERBB2 or NRG4 levels or both were low. These findings suggest a possible synergistic effect between these two prognostic factors. One possible mechanism is that in the presence of high ERBB2 levels, ERBB4 with bound NRG4 may form ERBB2:ERBB4 heterodimers, which possess oncogenic activity, rather than ERBB4:ERBB4 homodimers, which function as tumor suppressors [25,51]. Many NRG4 studies have been conducted in patients with non-alcoholic fatty liver disease and metabolic disease rather than in those with cancer. Serum NRG4 levels are decreased in patients with non-alcoholic fatty liver, acute coronary syndrome, and metabolic disorders compared to control groups [52,53,54], and upregulation of NRG4 attenuates insulin resistance and decreases hepatic steatosis [55,56]. Recently, the incidence of liver cirrhosis and HCC related to NASH has increased, and examining the role of NRG4 in the development and progression of HCC-related NASH is necessary.

Serum levels of MIG6, one of the four inducible feedback inhibitors of the activated EGFR signaling pathway, were also measured. MIG6 is particularly abundant in the liver [57], and decreased MIG6 expression is associated with poor prognosis in patients with HCC [58,59]. However, this study did not demonstrate a relationship between serum MIG6 levels and tumor characteristics, although serum levels of MIG6 were high in patients with PVTT and distant metastases.

Based on the results of this study, which showed a significant relationship between ERBB2, NRG4, and MIG6 and tumor characteristics, we further evaluated the predictive performance of these potential biomarkers. As a result, we demonstrated that the product of ERBB2 and NRG4, with or without MIG6, can consistently predict 6-month, 1-year, 3-year, and 5-year mortality better than AFP, especially for 6-month mortality. Since our study focused on the prognosis of HCC, controls such as healthy controls, chronic viral hepatitis B, or liver cirrhosis were not included, and the predictive performance of our marker using ERBB2, NRG4, and MIG6 for the diagnosis of HCC could not be confirmed. Therefore, further research should be conducted to validate whether these biomarkers could be applied for the early diagnosis of HCC using the serum marker we proposed in HCC patients compared to the other control group.

This study had several limitations. First, the serum levels of ERBB2, NRG4, and MIG6, not the tissue levels of these biomarkers, were measured. Therefore, determining whether the high concentration in the blood correlates to its expression in tumors was not possible. However, tissue expression levels in patients with advanced-stage HCC, which cannot be treated surgically and are diagnosed by imaging without biopsy, are difficult to evaluate. Furthermore, because of the heterogeneity of HCCs, some of the collected HCC tissues may not always correlate with the serum levels of biomarkers. Second, since a CR was achieved only in patients who received curative treatment, patients with advanced HCC or non-curative treatments, such as transarterial chemoembolization, were not included in the analyses of tumor recurrence. Third, confirming whether serum levels of ERBB2 and NRG4 were elevated in these patients was not possible because patients with germ cell tumors, colitis, and acute hepatitis, for which AFP is a false positive, were not included in this study. Fourth, this was a retrospective study; thus, selection bias may have existed when the samples were stored in a biobank. Therefore, based on the findings of this study, further prospective studies with a large sample size are necessary.

Despite the limitations, the strength of our study is that, to the best of our knowledge, it is the first to demonstrate the significance of serum ERBB2 and NRG4 levels as prognostic markers for HCC.

## 5. Conclusions

The combination of serum ERBB2, NRG4, and MIG6 levels could better predict mortality in patients with HCC than AFP. Further prospective studies are needed to examine whether serum ERBB2 and NRG4 levels can predict pretreatment response and evaluate the responses to treatments as well as validate them as prognostic markers in HCC.

## Figures and Tables

**Table 1:** Information pertaining to clinical and laboratory parameters of patients.

Characteristics	Total Patients (n = 60), n (%)
Male sex	47 (78.3)
Age, yrs	61.95 ± 9.87
HTN (present)	18 (30.0)
DM (present)	19 (31.7)
Chronic viral hepatitis (B/C)	42 (70.0)/7 (11.7)
Child–Pugh class (A/B/C)	48 (80.0)/8 (13.3)/4 (6.7)
Fatty liver (present)	10 (16.7)
Liver cirrhosis (present)	48 (80.0)
Tumor-related characteristics	
Maximal diameter, cm	3.99 ± 3.62
Number (single/multiple)	31 (51.7)/29 (48.3)
Portal vein tumor thrombus	10 (16.7)
Extrahepatic spread (present)	5 (8.3)
Tumor stage	
mUICC stage (I/II/III/IV)	15 (25.0)/16 (26.7)/14 (23.3)/15 (25.0)
BCLC stage (0/A/B/C/D)	14 (23.3)/20 (33.3)/7 (11.7)/15 (25.0)/4 (6.7)
Baseline laboratory findings	
AFP, ng/mL	11.51 (4.39–87.59) ^1^
Total bilirubin, mg/dL	1.19 ± 1.03
Prothrombin time, INR	1.12 ± 0.13
Albumin, mg/dL	1.19 ± 1.03
ERBB2, ng/mL	1.89 ± 0.96
NRG4, ng/mL	0.24 ± 0.19
MIG6, ng/mL	2.03 ± 4.66
Treatment modality	
Resection/RFA/TACE/RTx/CTx/BSC	25 (41.7)/21 (35.0)/2 (3.3)/0 (0.0)/5 (8.3)/7 (11.7)
Patients with previous treatment, total	16 (26.7)
Resection/RFA/TACE/RTx/CTx	2 (3.3)/2 (3.3)/11 (18.3)/1 (1.7)/0 (0)
Histopathological diagnosis	27 (45.0)

Values are presented as mean ± SD, except for ^1^ median (Q1–Q3). HTN, hypertension; DM, diabetes mellitus; mUICC, modified Union for International Cancer Control; BCLC, Barcelona Clinic Liver Cancer; AFP, alpha-fetoprotein; IQR, interquartile range; INR, international normalized ratio; ERBB2, erythroblastic leukemia viral oncogene homolog 2; NRG4, neuregulin 4; MIG6, mitogen-inducible gene 6; RFA, radiofrequency ablation; TACE, transcatheter arterial chemoembolization; RTx, radiotherapy; CTx, chemotherapy; BSC, best supportive care.

**Table 2:** The relationship between ERBB family-related proteins and tumor characteristics.

Characteristics	Variables	Value ^1^			*p*-Value	Spearman	*p*-Value
BCLC stage (0–A vs. B vs. C–D)		0–A (n = 34)	B (n = 7)	C–D (n = 19)			
	ERBB2	1.39 (1.00–2.58)	1.59 (1.47–2.10)	1.89 (1.72–3.42)	0.010	0.386	0.002
	NRG4	0.14 (0.10–0.19)	0.35 (0.33–0.44)	0.25 (0.18–0.43)	<0.001	0.609	<0.001
	MIG6	0.61 (0.36–1.36)	1.19 (0.82–3.39)	1.29 (0.61–2.81)	0.046	0.281	0.030
Tumor size (<2 cm vs. 2–5 cm vs. >5 cm)		<2 cm (n = 25)	2–5 cm (n = 19)	>5 cm (n = 16)			
	ERBB2	1.36 (0.73–1.85)	1.73 (1.42–2.54)	1.87 (1.60–3.42)	0.004	0.432	0.001
	NRG4	0.16 (0.12–0.24)	0.20 (0.12–0.33)	0.21 (0.16–0.40)	0.253	0.212	0.104
	MIG6	0.64 (0.42–1.41)	0.61 (0.17–1.91)	1.24 (0.75–2.62)	0.168	0.205	0.115
Number of tumors (single vs. 2–3 vs. >3)		1 (n = 31)	2–3 (n = 21)	>3 (n = 8)			
	ERBB2	1.39 (1.06–2.00)	1.86 (1.45–3.02)	1.81 (1.53–3.01)	0.055	0.304	0.018
	NRG4	0.15 (0.10–0.19)	0.26 (0.17–0.43)	0.29 (0.21–0.41)	<0.001	0.558	<0.001
	MIG6	0.64 (0.40–1.36)	1.10 (0.49–2.81)	1.37 (0.91–2.47)	0.186	0.232	0.074

^1^ Values are presented as median (Q1–Q3). ERBB family-related proteins, include ERBB2, NRG4, and MIG6; ERBB2, erythroblastic leukemia viral oncogene homolog 2; NRG4, neuregulin 4; MIG6, mitogen-inducible gene 6.

**Table 3:** The relationship between ERBB family-related proteins and portal vein tumor thrombus, distant metastasis, liver cirrhosis, and hepatitis B virus infection.

Characteristics	Variables	Value ^1^		*p*-Value
Portal vein tumor thrombus (absent vs. present)		Absent (n = 50)	Present (n = 10)	
	ERBB2	1.50 (1.13–2.12)	2.65 (1.86–3.61)	0.001
	NRG4	0.17 (0.12–0.25)	0.32 (0.20–0.55)	0.007
	MIG6	0.65 (0.38–1.36)	2.00 (1.12–3.75)	0.004
Distant metastasis (absent vs. present)		Absent (n = 55)	Present (n = 5)	
	ERBB2	1.61 (1.17–2.54)	1.85 (1.68–4.21)	0.093
	NRG4	0.18 (0.12–0.31)	0.25 (0.23–0.45)	0.052
	MIG6	0.66 (0.45–1.45)	2.81 (1.08–4.21)	0.023
Liver cirrhosis (absent vs. present)		Absent (n = 12)	Present (n = 48)	
	ERBB2	2.71 (1.44–3.25)	1.62 (1.19–1.98)	0.065
	NRG4	0.17 (0.08–0.24)	0.19 (0.13–0.32)	0.144
	MIG6	1.01 (0.54–1.38)	0.82 (0.41–1.87)	0.882
Chronic viral hepatitis B (absent vs. present)		Absent (n = 18)	Present (n = 42)	
	ERBB2	1.63 (1.14–1.86)	1.76 (1.30–2.77)	0.583
	NRG4	0.25 (0.20–0.35)	0.16 (0.12–0.24)	0.003
	MIG6	1.56 (0.57–3.54)	0.66 (0.46–1.23)	0.018
Chronic viral hepatitis C (absent vs. present)		Absent (n = 53)	Present (n = 7)	
	ERBB2	1.80 (1.28–2.73)	1.47 (1.06–1.64)	0.210
	NRG4	0.18 (0.12–0.26)	0.31 (0.20–0.43)	0.093
	MIG6	0.66 (0.46–1.38)	2.81 (1.37–3.95)	0.012
Fatty liver (absent vs. present)		Absent (n = 50)	Present (n = 10)	
	ERBB2	1.67 (1.30–2.27)	1.75 (0.72–3.23)	0.905
	NRG4	0.19 (0.14–0.32)	0.14 (0.09–0.28)	0.275
	MIG6	0.77 (0.46–1.58)	1.13 (0.55–2.98)	0.427

^1^ Values are presented as median (Q1–Q3). ERBB family-related proteins, include ERBB2, NRG4, and MIG6; ERBB2, erythroblastic leukemia viral oncogene homolog 2; NRG4, neuregulin 4; MIG6, mitogen-inducible gene 6.

**Table 4:** Univariate and multivariate analysis of factors predicting overall survival.

Variables	Univariate Analysis			Multivariate Analysis		
	HR	95% CI	*p*-Value	Adjusted HR	95% CI	*p*-Value
Age	1.019	0.971–1.070	0.442			
Sex (female)	0.892	0.298–2.670	0.838			
DM	2.058	0.848–4.994	0.110			
Chronic viral hepatitis B	0.355	0.147–0.859	0.022	0.543	0.181–1.626	0.267
Liver cirrhosis	1.873	0.431–8.144	0.402			
Child–Pugh (B and C)	8.563	3.476–21.094	<0.001	4.936	1.219–19.990	0.025
Maximal tumor diameter	1.237	1.109–1.380	<0.001	0.993	0.791–1.246	0.959
BCLC stage (C and D)	11.657	4.005–33.930	<0.001	6.523	1.704–24.965	0.006
AFP (ng/mL)	1.000	1.000–1.001	0.022	1.001	1.000–1.001	0.017
HCC treatment						
Resection or RFA	1	reference				
TACE or CTx	6.314	1.907–20.901	0.003	1.117	0.046–27.255	0.488
Supportive care	26.355	7.393–93.951	<0.001	2.794	0.065–120.549	0.372
ERBB2 (ng/mL)	2.959	1.788–4.896	<0.001	2.719	1.317–5.613	0.007
NRG4 (ng/mL)	23.924	3.970–144.158	0.001	14.347	0.681–302.105	0.077
MIG6 (ng/mL)	1.023	0.958–1.093	0.490			

DM, diabetes mellitus; BCLC, Barcelona Clinic Liver Cancer; AFP, alpha-fetoprotein; RFA, radiofrequency ablation; TACE, transcatheter arterial chemoembolization; CTx, chemotherapy; ERBB2, erythroblastic leukemia viral oncogene homolog 2; NRG4, neuregulin 4; MIG6, mitogen-inducible gene 6; CI, confidence interval.

**Table 5:** Univariate and multivariate analyses of factors predicting HCC recurrence.

Variables	Univariate Analysis			Multivariate Analysis		
	HR	95% CI	*p*-Value	Adjusted HR	95% CI	*p*-Value
Age	1.018	0.976–1.063	0.407			
Sex (female)	0.716	0.271–1.888	0.499			
DM	1.429	0.644–3.170	0.380			
Chronic viral hepatitis B	0.867	0.367–2.049	0.744			
Liver cirrhosis	0.910	0.366–2.267	0.840			
Child–Pugh (B and C)	0.855	0.200–3.648	0.832			
Maximal tumor diameter	1.128	0.974–1.306	0.108			
BCLC stage (C and D)	2.845	0.966–8.377	0.058	2.535	0.844–7.611	0.086
AFP (ng/mL)	1.001	1.000–1.002	0.104			
HCC treatment						
Resection	1	reference				
RFA	0.968	0.460–2.039	0.933			
ERBB2 (ng/mL)	1.756	1.106–2.787	0.017	2.338	1.350–4.050	0.002
NRG4 (ng/mL)	161.842	8.941–2929.485	0.001	431.763	19.417–9600.648	<0.001
MIG6 (ng/mL)	1.006	0.945–1.071	0.846			

DM, diabetes mellitus; BCLC, Barcelona Clinic Liver Cancer; AFP, alpha-fetoprotein; RFA, radiofrequency ablation; ERBB2, erythroblastic leukemia viral oncogene homolog 2; NRG4, neuregulin 4; MIG6, mitogen-inducible gene 6; CI, confidence interval.

**Table 6:** ROC curves for ERBB family-related proteins, combinations of ERBB family-related proteins, and serum AFP for predicting 6-month mortality.

Variable(s)	Cut-Off	Sensitivity (%)	Specificity (%)	AUC (CI 95%)	*p*-Value
AFP (ng/mL)	17.9	77.78	68.18	0.727 (0.502–0.952)	0.0476
MIG6 (ng/mL)	0.9135	100.00	66.00	0.844 (0.727–0.925)	<0.0001
NRG4 (ng/mL)	0.2107	100.00	70.00	0.888 (0.780–0.955)	<0.0001
ERBB2 (ng/mL)	3.2552	60.00	100.00	0.850 (0.734–0.929)	<0.0001
ERBB2 × NRG4	0.4636	90.00	84.00	0.942 (0.850–0.986)	<0.0001
ERBB2 × MIG6	3.8697	100.00	88.00	0.940 (0.847–0.985)	<0.0001
NRG4 × MIG6	0.2863	100.00	80.00	0.900 (0.795–0.962)	<0.0001
ERBB2 × NRG4 × MIG6	0.8430	100.00	90.00	0.940 (0.847–0.985)	<0.0001

Abbreviations: ROC, receiver operating characteristic; ERBB family-related proteins, including ERBB2, NRG4, and MIG6; AFP, alpha-fetoprotein; ERBB2, erythroblastic leukemia viral oncogene homolog 2; NRG4, neuregulin 4; MIG6, mitogen-inducible gene 6; AUC, area under the curve; CI, confidence interval.

## Data Availability

The data used and/or analyzed during the current study are available from the corresponding author upon reasonable request.

## Supplementary Materials

The following supporting information can be downloaded at: https://www.mdpi.com/article/10.3390/cancers15092634/s1, Table S1: Receiver operating characteristic curves for ERBB family-related proteins, combinations of ERBB family-related proteins, and serum AFP for predicting 1-year, 3-year, and 5-year mortality, Figure S1: Receiver operating characteristic curves for ERBB family-related proteins and combinations of ERBB family-related proteins for the prediction of 1-year, 3-year, and 5-year mortality.
