# Supplementary material for: Prognostic Value of Erythroblastic Leukemia Viral Oncogene Homolog 2 and Neuregulin 4 in Hepatocellular Carcinoma
Source: Cancers (Basel). 2023 May 6;15(9):2634. doi: 10.3390/cancers15092634 (PMC10177431; doi:10.3390/cancers15092634)
Supplement: Supplementary file 1 [file cancers-15-02634-s001.zip › cancers-2351308-supplementary.pdf]

Supplementary Materials

Table S1. ROC curves for ERBB family-related proteins, combinations of ERBB family-related proteins, and serum AFP for predicting 1-year, 3-year, and 5-year mortality.

Variables	Cut-off	Sensitivity (%)	Specificity (%)	AUC (CI 95%)	*p*-Value
1-year mortality					
AFP (ng/mL)	17.9	70.00	67.44	0.656 (0.513–0.781)	0.2078
MIG6 (ng/mL)	0.9135	90.91	65.31	0.767 (0.640–0.867)	0.0026
NRG4 (ng/mL)	0.2107	90.91	69.39	0.857 (0.743–0.934)	< 0.0001
ERBB2 (ng/mL)	1.6117	100.00	57.14	0.844 (0.727–0.925)	< 0.0001
ERBB2×NRG4	0.3388	100.00	69.39	0.918 (0.818–0.973)	< 0.0001
ERBB2×MIG6	3.8697	90.91	87.76	0.856 (0.742–0.933)	< 0.0001
NRG4×MIG6	0.2863	90.91	79.59	0.819 (0.698–0.907)	0.0002
ERBB2×NRG4×MIG6	0.8430	90.91	89.80	0.856 (0.742–0.933)	< 0.0001
3-year mortality					
AFP (ng/mL)	17.9	68.75	72.97	0.667 (0.524–0.791)	0.0628
MIG6 (ng/mL)	0.9135	82.35	69.77	0.779 (0.653–0.876)	0.0001
NRG4 (ng/mL)	0.1968	76.47	67.44	0.778 (0.653–0.875)	< 0.0001
ERBB2 (ng/mL)	1.4201	94.12	46.51	0.740 (0.611–0.845)	0.0006
ERBB2×NRG4	0.3388	82.4	72.1	0.828 (0.708–0.913)	< 0.0001
ERBB2×MIG6	2.3728	76.47	81.40	0.817 (0.696–0.905)	< 0.0001
NRG4×MIG6	0.1299	94.12	67.44	0.832 (0.714–0.916)	< 0.0001
ERBB2×NRG4×MIG6	4.4991	94.12	65.12	0.827 (0.707–0.912)	< 0.0001
5-year mortality					
AFP (ng/mL)	17.9	57.89	70.59	0.607 (0.463–0.738)	0.2249
MIG6 (ng/mL)	0.6573	85.00	60.00	0.733 (0.603–0.839)	0.0014
NRG4 (ng/mL)	0.2361	65.00	82.50	0.789 (0.664–0.883)	< 0.0001
ERBB2 (ng/mL)	1.6117	80.00	60.00	0.736 (0.607–0.842)	0.0004
ERBB2×NRG4	0.4309	70.00	87.50	0.831 (0.712–0.916)	< 0.0001
ERBB2×MIG6	1.9115	75.00	72.50	0.773 (0.647–0.871)	0.0001
NRG4×MIG6	0.2863	70.00	85.00	0.804 (0.682–0.895)	< 0.0001
ERBB2×NRG4×MIG6	0.6622	70.00	92.50	0.818 (0.697–0.906)	< 0.0001

ROC, receiver operating characteristic; ERBB family-related proteins, include ERBB2, NRG4, and MIG6; AFP, alpha-fetoprotein; ERBB2, erythroblastic leukemia viral oncogene homolog 2; NRG4, neuregulin 4; MIG6, mitogen-inducible gene 6; AUC, area under the curve; CI, confidence interval.

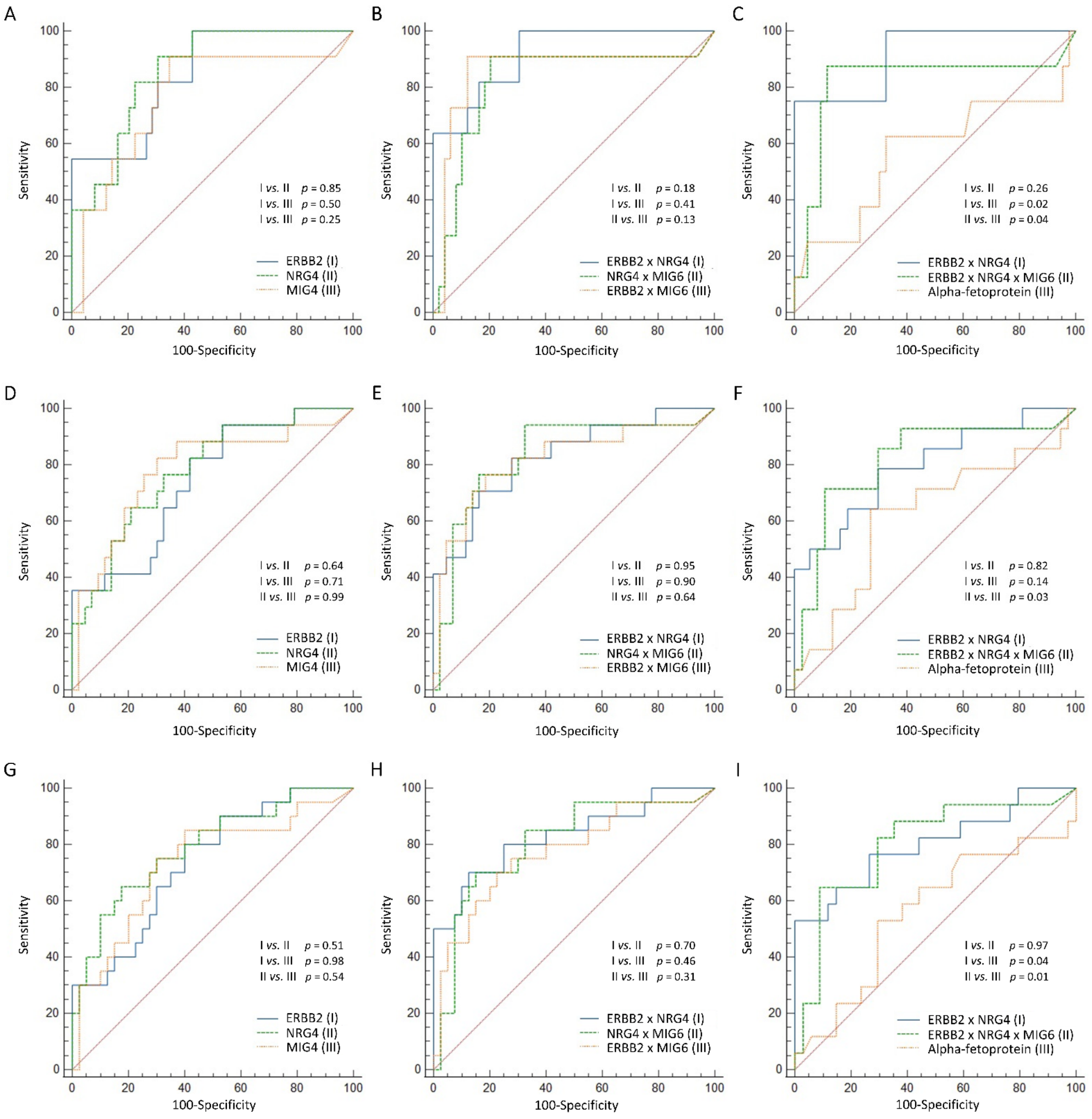

Figure S1. Graphs A-C, D-F, and G-I are receiver operating characteristic curves for the prediction of 1-year, 3-year, and 5-year mortality, respectively. (A, D, G) The single factors, such as ERBB2, NRG4, MIG6, were compared to each other and (B, E, H) multiplied values were also compared. The product of ERBB2 and NRG4 with/without MIG6 showed the best result in the area under AUC. (C, F, I) This product shows superior results in AUC, sensitivity, and specificity, compared to the results of alpha-fetoprotein. (ERBB2, erythroblastic leukemia viral oncogene homolog 2; NRG4, neuregulin 4; MIG6, mitogen-inducible gene 6; AUC, area under the curve).
